# Application of UiO-66 MOF for rifampicin removal and post-adsorption antimicrobial activity against MRSA

**DOI:** 10.1038/s41598-025-10145-4

**Published:** 2025-07-16

**Authors:** S. A. Abdel Moaty, Amna A. Kotp, Asmaa M. Salah, Ahmed A. Farghali, Zienab E. Eldin

**Affiliations:** 1 Chemistry Department, Faculty of Science, Beni-Suef University, Beni-Suef, Egypt; 2 Materials Science and Nanotechnology Department, Faculty of Postgraduate Studies for Advanced Sciences, Beni-Suef University, Beni-Suef, 62511 Egypt

**Keywords:** UiO-66, Rifampicin adsorption, Antibacterial activity, MRSA, Biocompatibility, Drug delivery., Microbiology, Chemistry, Materials science, Nanoscience and technology

## Abstract

Water is essential to all living forms, shaping both our planet and the human body. But their great toxicity makes dangerous contaminants like new chemicals, antibiotics, heavy metals, and microbes major dangers to water systems. This article looks at the creation and use of UiO-66, a zirconium-based metal-organic framework (MOF), as a dual-function material for rifampicin adsorption and antibacterial action. Using solvothermal techniques, UiO-66 MOFs were created and characterized by X-ray diffraction (XRD), nitrogen adsorption-desorption, field-emission scanning electron microscopy (FESEM), thermogravimetric analysis (TG), Fourier-transform infrared spectroscopy (FTIR), and Brunauer–Emmett-Teller (BET) analysis. Batch testing maximized factors like pH, starting concentration, adsorbent amount, and contact time to improve rifampicin removal effectiveness. The findings showed a maximum adsorption capacity (q_max_) of 542 mg/g under ideal circumstances, suggesting UiO-66 MOF as a reasonably priced and sustainable choice for treating contaminated wastewater. Further research confirmed UiO-66’s potential as a good adsorbent for rifampicin under different settings. At lower doses, UiO-66 nanoparticle cytotoxicity on HL-7702 liver cells revealed great biocompatibility; at higher levels, it caused significant viability loss. The antibacterial effectiveness of UiO-66 MOF nanoparticles, rifampicin, and their combination against Klebsiella pneumoniae and MRSA was assessed; the combination greatly boosted antibacterial activity in comparison to separate therapies. The biocompatibility of the composite with human cells and capacity to damage bacterial cell membranes point to its possible use as an antibacterial and medication delivery system. The research offers a sustainable environmental remediation option by demonstrating that UiO-66, a zirconium-based MOF, efficiently adsorbs rifampicin from wastewater. Its high adsorption capacity of 542 mg/g points to possible treatment of MRSA and K. pneumoniae. See Fig. 1 for more details. Future studies should include property optimization, industrial application scalability, and drug interaction research.

## Introduction

Water pollution is a critical environmental issue in the twentyfirst century due to hazardous substances released by human activities. Among these contaminants, antibiotics are particularly concerning because of their persistent presence in drinking, surface, and wastewater. This has led to the development of undesirable products in water, the generation of microbial resistance genes, and the destruction of aquatic life^1,2^.

**Fig. 1:**
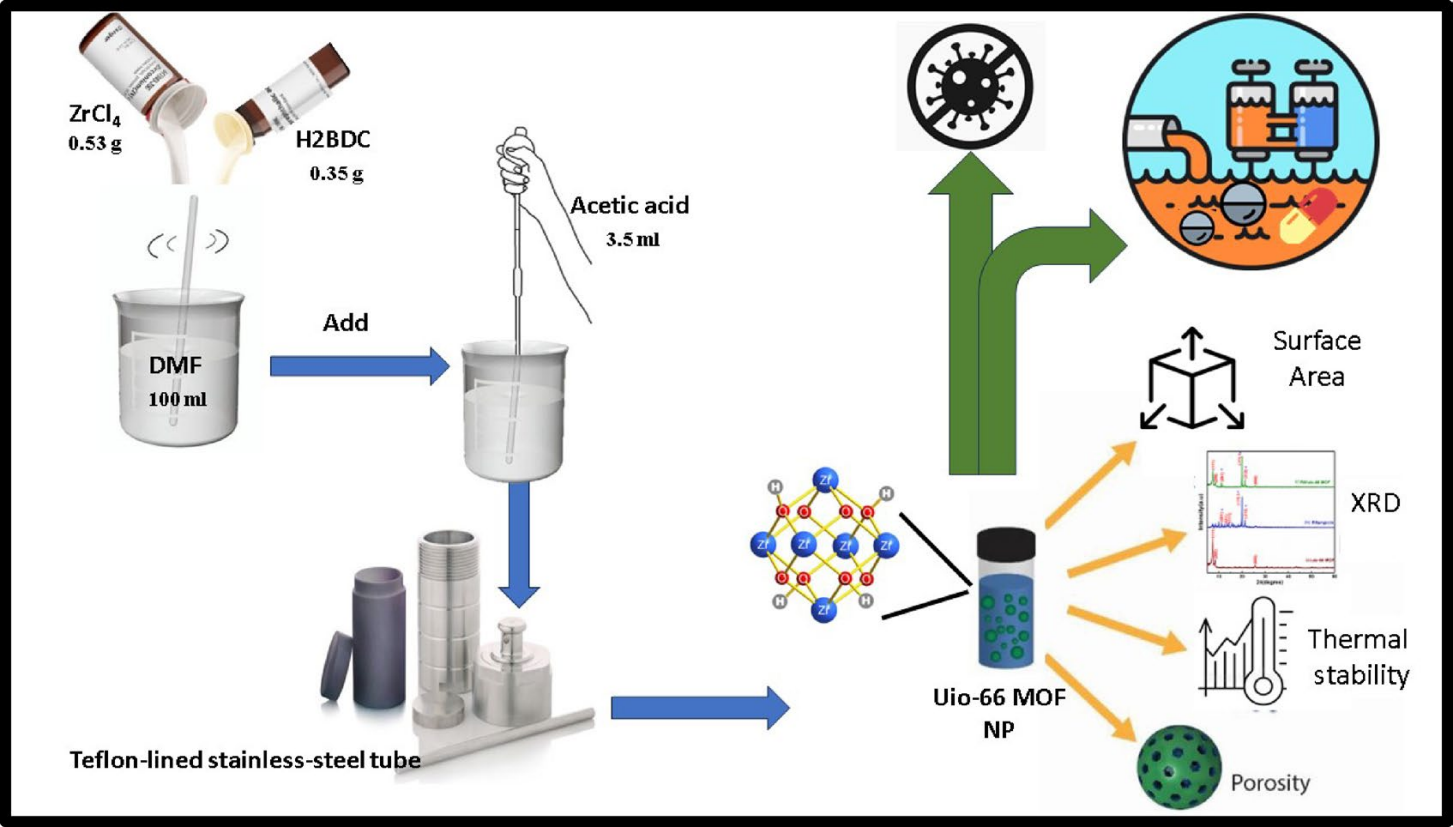
The procedure of synthesis of the UiO-66 MOF NP illustrating its significant role in watertreatment and antimicrobial activity.

The overuse of antibiotics in aquaculture, livestock, and medical industries leads to water pollution, as limited bioavailability can prolong antibiotic use in the gastrointestinal tract and release unabsorbed antibiotics into the environment, causing biological effects. The rise of antibiotic-resistant bacteria like MRSA poses a significant public health risk^1,2^. Antibiotics are becoming ineffective against resistant bacteria, necessitating novel methods like adsorption technologies for environmental remediation due to their efficiency, cost-effectiveness, minimal byproduct formation, and low energy requirements (Fig. 1).

Nanotechnology and environmental consciousness have led to the widespread use of absorbent materials like activated carbon, zeolite, and metal oxides, offering high efficiency, low cost, and environmental friendliness, while Metalorganic Frameworks (MOFs) are organic-inorganic hybrids^3^. Unsaturated metal vacancies and varying pore sizes and volumes can be utilized to create functional adsorption materials using metal active centers or specific-sized hole structures^4^.

Metal-organic frameworks (MOFs) are attractive materials for drug delivery and antibacterial use due to their tunable porosity, large surface area, and diverse activity^4^. These materials are stable, can be modified with functional groups, and have simple synthesis techniques^5,6^. They are also compatible with Green Biomaterials principles, making their use as adsorbent materials sensible. Their versatility and compatibility with Green Biomaterials principles make them a promising option for various applications^7^.

Recent studies have shown the potential of Metal Organic Frame Work (MOFs) in medication delivery systems, such as thin films and MIL-based delivery systems for cancer treatment^8^. MOFs have shown improved performance in therapeutic settings^9^ and have shown utility in controlled release of antibiotics and anticancer medications^10^^11^. Recent developments have also focused on their biocompatibility and efficiency in drug administration^12^, and their potential in combination treatments.

While previous studies have demonstrated the effectiveness of various MOFs^13^ in antibiotic adsorption and antimicrobial activity^14–16^, our study focuses on the unique properties of UiO-66, a zirconium-based MOF, which offers several advantages over other MOFs. UiO-66 is known for its exceptional chemical and thermal stability, which enables it to retain its structural integrity under harsh environmental conditions, including exposure to various solvents and elevated temperatures. Moreover, its high surface area offers extensive active sites for drug loading, while its tunable porosity allows precise control over pore dimensions. These features are particularly advantageous for optimizing both the adsorption capacity and the sustained release profile of therapeutic agents, making UiO-66 an excellent candidate for drug delivery and adsorption applications^17^.

In comparison to other MOFs^4^, UiO-66 has demonstrated superior adsorption capacity for rifampicin, with a maximum adsorption capacity (q_max_) of 542 mg/g under optimal conditions. As illustrated in Table 1. Additionally, UiO-66 exhibits enhanced antibacterial activity against resistant bacteria such as Klebsiella pneumoniae and MRSA, making it a promising material for both environmental remediation and biomedical applications.

Furthermore, our study explores the cytotoxicity of the rifampicin-loaded UiO-66 MOF composite to confirm its compatibility with human cells, providing a comprehensive evaluation of its potential for drug delivery applications. By investigating the underlying antimicrobial mechanisms, we aim to gain a thorough understanding of how this innovative composite material can disrupt bacterial function and address the rising issue of antibiotic resistance. This study not only demonstrates the promise of UiO-66 in therapeutic applications but also provides important insights into the design of new materials for battling resistant bacterial strains.

**Table 1:** Previous studies in the open literature report adsorbents for rifampicin removal.

Adsorbent	pH	Equilibrium time	q_max_ (mg/g)	Ref.
UiO-66 MOF	5	180 min	542	This work
Fe_3_**O**_**4**_ Excoecaria cochinchinensis leaf extract	5.5	60 min	84.8	^18^
Fe NPs tea extract	3	120 min	107.7	^19^
Raw kaolin	–	250 min	5.931	^20^
Alkali-activated kaolin	–	250 min	8.295	^20^
Ce–Py–MSK	–	60 min	102.25	^21^
Activated carbon of cocoa shells	6	120 min	26.66	^22^
Calcined Mytella falcata shells	7	60	9.731	^23^
Sisal–Fe/Zn bionanocomposites	–	–	40.0	^24^
Chitosan		150	66.9	^25^

## Experimental

### Materials and reagents

Polyvinylpyrrolidone (PVP) (95%), 1, 4-benzenedicarboxylic acid (H_2_BDC) (99%), and zirconium chloride (ZrCl_4_) (99.5%) were acquired from Chem-Lab (Belgium). Rifampicin (99%) and p-nitrophenyl palmitate (pNPP; C16) were obtained from Sigma-Aldrich (USA), whilst Merck KGaA (Germany) provided N,N-dimethylformamide (DMF) (99.5%), glacial acetic acid, and dimethyl sulfoxide (DMSO) (99%).

### Preparation of UiO-66 nanoparticles

The synthesis of UiO-66 MOF was performed by the solvothermal strategy as reported earlier in^26^ with modification. Briefly, a mixture of ZrCl_4_ (0.53 g) and H_2_BDC (0.35 g) was dissolved in 100 mL of DMF at room temperature. Subsequently, 3.5 mL of glacial acetic acid was added and the resulting mixture was poured into a Teflon-lined stainless-steel tube, which was autoclaved at 121 °C for 24 h. Crystallization Nanoparticle production was carried out under static conditions. After the resulting mixture was cooled to room temperature, it was centrifuged to obtain UiO-66 MOF, then the precipitate was washed more than two times with DMF, dried in an oven at 150 °C for 24 h.

### Characterization of UiO-66 MOF

The as synthesize catalysts has been characterized by several tools. For example, the crystallinity and structure of the synthesized materials were measured with an Empyrean Panalytical X-ray diffractometer that used Cu–K radiation (wavelength 0.154 nm) and ran at 40 kV of voltage and 35 mA of current. After combining 150 mg of optically high-purity KBr with 0.25 mg of the obtained material in an agate mortar, the mixture was vacuum-sealed for five minutes. Next, the KBr pellet was crushed at 10 tons of pressure for fifteen minutes. As a consequence, the Zeiss standard KBr pellet has an area of 1.13 cm^2^. Furthermore, BET (TriStar II 3020, Micrometric, USA) was used to determine the specific pore volume, surface area, and pore size distribution of the produced samples. The microstructure was studied by a JSM-5410 scanning electron microscope (SEM). Following obtaining a light grey transparent pellet devoid of granules, the IR spectra were recorded at 4000 –400 cm-1 wave number range (FESEM) using a Bruker-Vertex 70, Germany, spectrometer. The DTA-TGA 2000 model from MAC-Science was utilized for doing the thermal study.

### Adsorption study

Rifampicin’s adsorption performance was assessed using the batch adsorption method in this work on UiO-66 MOF. Stock standard solutions of rifampicin were produced at a concentration of 1000 µg/ml; successive dilutions were performed to create calibration curves. Among the many factors that greatly influence the adsorption process are the amount of adsorbent, pH, concentrations of drugs, and contact time between the adsorbent and contaminants. pH was studied by inserting 0.05 g of the produced adsorbent in five conical flasks (50 ml each) and adding 50 ml of a 10 ppm rifampicin solution to every flask. Using 0.1 N NaOH or 0.1 N HCl, the pH of the solutions was set to 3, 5, 6, 7, and 8. To ensure complete mixing and interaction between the adsorbent and the drugs molecules, the flasks were shaking overnight on an orbital shaker at 180 rpm. The quantity of adsorbent was next investigated by adding several doses—0.025 g, 0.05 g, 0.075 g, 0.1 g, and 0.2 g-to extra falcon tubes set at the optimal pH found in the prior stage. This stage sought to find the best adsorbent dose maximizing rifampicin adsorption and minimum material use. Under the best conditions found from the prior phases, a range of 5–500 g/ml was studied to see how drug concentration affected. Covering low to high amounts of rifampicin, this spectrum simulates various degrees of wastewater pollution.

Under the best conditions gained from the prior phases, the influence of contact time was finally investigated. To establish the kinetics of rifampicin adsorption onto UiO-66 MOF, many contact durations were examined, hence shedding light on the rate and efficiency of the adsorption process. To better understand the adsorption kinetics and efficiency, the interactions between rifampicin and UiO-66 MOF were tracked throughout time. The residual concentration of rifampicin in the solution after the adsorption procedure determined the adsorption capacity. This thorough study gave a complete knowledge of the drug-MOF interactions and let one find ideal conditions for rifampicin elimination.

Tests on recyclability assessed UiO-66 MOF performance after many adsorption cycles. The findings showed a slow drop in removal efficiency across numerous cycles, implying a loss in adsorptive ability. Partial pore obstruction, structural deterioration, inadequate rifampicin desorption, and adsorbate-induced surface chemistry alterations are among the factors causing this decline. Used as a desorption agent, ethanol has varying impacts on the MOF’s performance and structural integrity. Although ethanol first successfully eliminated adsorbed rifampicin, its effectiveness dropped with each cycle because it could not reach deeply adsorbed molecules or desorb rifampicin interacting strongly with the MOF surface. Solvent-induced degradation might have minor swelling or structural alterations to the UiO-66 framework, hence compromising its stability and adsorptive potential. Though efficiency dropped, UiO-66 maintained a fair adsorption capacity (70%) after five cycles, hence it was a reasonably priced choice for uses where some efficiency loss is tolerable. Alternative desorption processes, functionalizing or coating UiO-66 MOF, and improving regeneration techniques were investigated to increase recyclability. Adding hydrophilic or hydrophobic groups to the surface chemistry of the MOF and refining regeneration methods like thermal treatment or supercritical fluids might perhaps restore the structure and adsorption capacity of the MOF more effectively than ethanol, therefore reducing the efficiency loss over many cycles.

### Cytotoxicity effect (MTT assay)

The normal hepatic HL-7702 cell line was acquired from the tissue culture unit of the Holding Company for Biological Products and Vaccines (VACSERA). These cells were cultured in DMEM growth medium supplemented with antibiotics (0.9% saline containing 20 mg amoxicillin and 25 mg chloramphenicol) and 10% phosphate-buffered saline (PBS). Cells were plated at a density of 1 × 10^4^ cells per well (100 µL) and incubated at 37 °C in a 5% CO_2_ atmosphere for 24 h. After the cells had adhered, they were exposed to various concentrations of UiO-66, ranging from 1000 to 6.25 µg/mL. Following this, 10 µL of a 12-mM MTT stock solution (prepared by dissolving 5 mg/mL MTT in sterile PBS) was added to each well. The cells were then incubated for an additional 4 h at 37 °C, after which the MTT solution was removed^27^.

In addition to the HL-7702 hepatic cell line, we added human kidney epithelial cells (HK-2) and human lung fibroblast cells (MRC-5). Human kidney epithelial cells (HK-2) and human lung fibroblast cells (MRC-5) were obtained from (Vacsera, Cairo, Egypt).

These extra cell lines were grown under comparable circumstances and subjected to the same spectrum of UiO-66 concentrations. This wider study guarantees the biocompatibility of UiO-66 MOF across many cell types, hence confirming its appropriateness for biomedical uses.

The investigation on UiO-66 nanoparticles and their impact on HL-7702 liver cells shows a concentration-dependent cytotoxicity. Cell survival stays almost 100% at lower dosages (7.8 to 62.5 µg/ml), suggesting little cytotoxicity and strong biocompatibility. Viability shows a little drop as the concentration rises to 125 g/ml; more noticeable drops occur at 250 g/ml (about 80.12% viability) and 500 g/ml (around 77% viability). Cell survival is around 72% at the maximum dose of 1000 g/ml. These results imply that while UiO-66 nanoparticles are usually safe at low concentrations, greater dosages might have significant cytotoxic consequences, hence stressing the need of cautious dosing in possible biomedical and water treatment uses.

### Estimation of the antimicrobial activity

#### Bacterial strains and growth conditions

The study utilized the following bacterial strains: Klebsiella pneumoniae (ATCC: 13882) and methicillin-resistant Staphylococcus aureus (MRSA; ATCC: 43300. Klebsiella pneumoniae was cultured or maintained on tryptic soy agar/broth (TSA or TSB) and MacConkey agar (Oxoid, UK). MRSA was grown on brain heart infusion (BHI) agar/broth (Oxoid) with the addition of 1% glucose and sucrose, respectively. Both strains were incubated aerobically at 37 °C for 24 h.

#### Minimum inhibitory concentration (MIC)

The antimicrobial efficacy of Rif/UiO-66 MOF was evaluated using the broth microdilution method in a 96-well plate format. The standard drug and its nanoparticle formulations were subjected to serial two-fold dilutions in Mueller Hinton broth (Oxoid). Each bacterial strain was then added to its respective wells at a concentration of 5 × 10⁵ CFU/mL. The plate included vehicle control (1% DMSO), positive control, and negative control wells for each bacterial lane. After incubation for 24 h at 37 °C, the minimum inhibitory concentration (MIC) was determined. The MIC was defined as the lowest concentration of the compound that inhibited visible bacterial growth.

#### Minimal bactericidal concentration (MBC)

The Minimum Bactericidal Concentration (MBC) is defined as the lowest concentration of a substance that prevents the formation of visible bacterial colonies after a 24-h incubation period at 37 °C. This measurement indicates the substance’s capacity to effectively eliminate bacteria.

#### Statistical analysis

The data were analyzed using ANOVA, which included descriptive statistics like mean and standard deviation. Tukey’s post hoc test was used to examine MIC and MBC, with all statistical tests deemed significant at *p* < 0.05.

## Results and discussion

### The characterization of catalyst

**Fig. 2:**
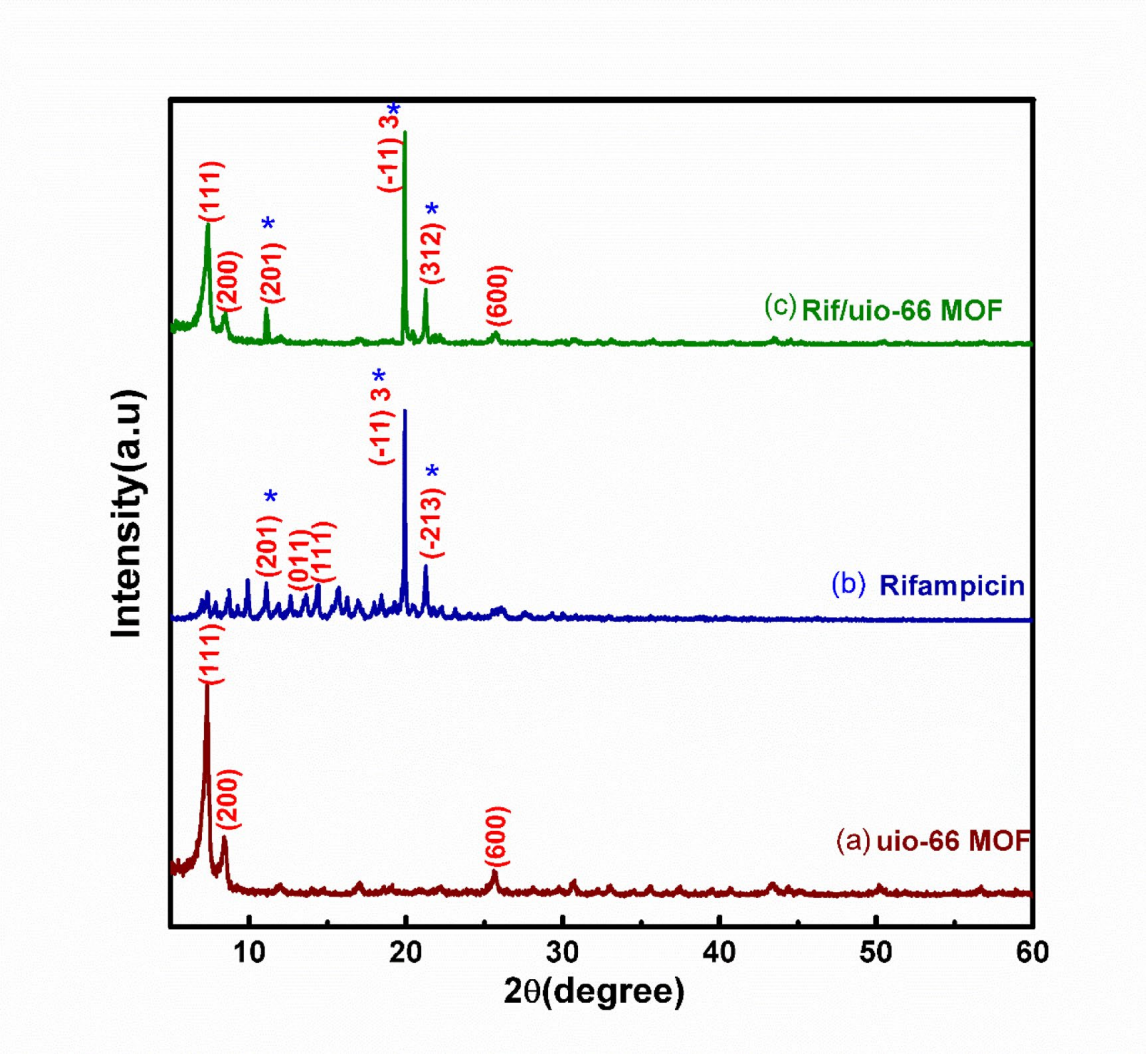
**.** XRD analysis of (**a**) UiO- 66 MOF, (**b**) Rifampicin, and (**c**) Rif/UiO-66 MOF.

The X-ray diffraction (XRD) study shows that when loaded with rifampicin the UiO-66 MOF preserves its crystalline structure, which is crucial for providing the MOF’s capacity to keep and release the drug efficiently. The XRD patterns of the produced samples before and after rifampicin inclusion are shown in Fig. 2a. Characteristic peaks for UiO-66 MOF show at (2θ = 7.14°, 8.56° and 25.42°), respectively corresponding to the (111), (002), and (006) planes^9,28^. The preservation of rifampicin’s crystalline peaks suggests that the drug is included in a that maintains its structural integrity, which is crucial for its therapeutic effectiveness. Exhibiting diffraction peaks at (2θ = 11.903°, 13.704° 14.400°, 18.451°, and 21.275°), Fig. 2b displays the XRD patterns of rifampicin assigned to the (201), (011), (111), (-113), and (-213) planes, respectively. Both UiO-66 MOF and rifampicin show great crystallinity as shown by the strong and distinct peaks in their XRD patterns. In drug delivery applications, this crystallinity improves the stability and performance of the composite.

The XRD pattern of the composite, seen in Fig. 2c, suggests efficient synthesis as both the MOF and drug retain their structural identities while creating a new material with perhaps unique properties. The extra peaks in the composite pattern point to interactions between the MOF and rifampicin, which call for further research to clarify the behavior of the composite.

Valuable insights on the structural characteristics and interactions of UiO-66 MOF, rifampicin, and their composite (Rif/UiO-66 MOF) are provided by XRD study. While preserving the structural qualities of both the MOF and the drug, the maintenance of crystallinity and the appearance of new peaks in the composite suggest effective synthesis. For drug delivery uses, where controlled release and preservation of the drug’s efficacy depend on structural integrity, this is favorable.

**Fig. 3:**
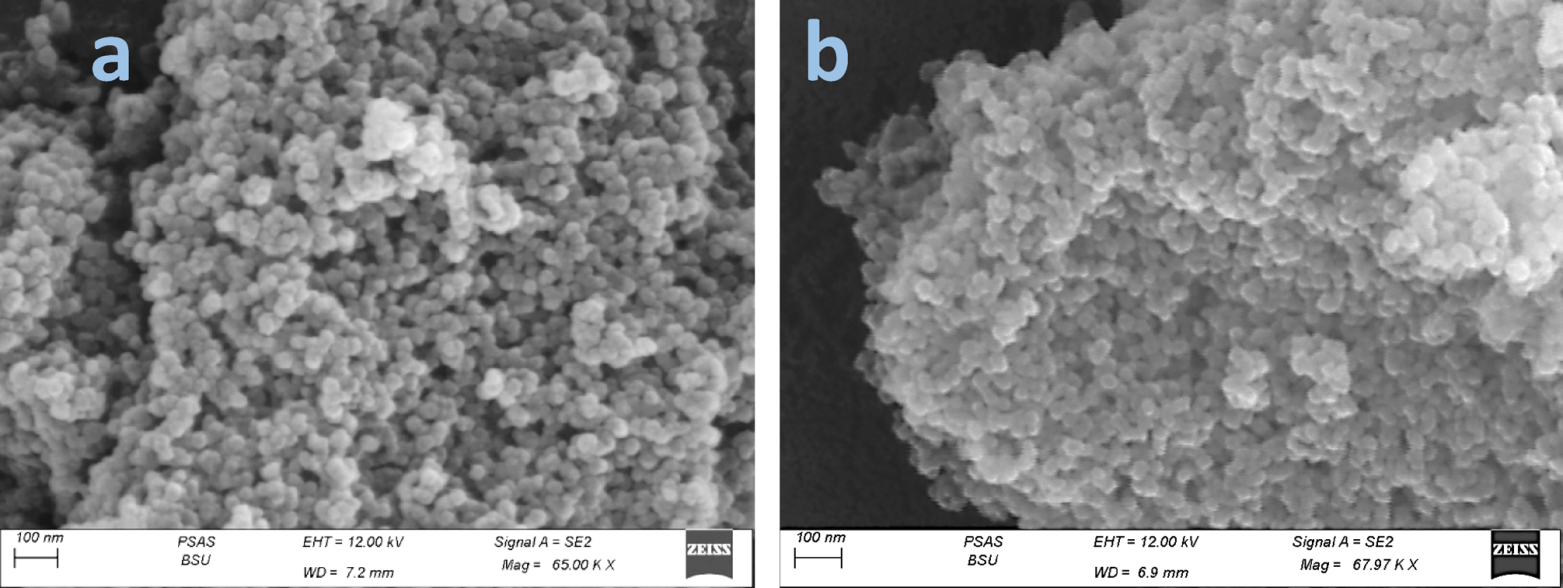
SEM images of (**a**) UiO- 66 MOF, (**b**) Rif/UiO-66 MOF.

Figure 3. Show The SEM micrographs provide a clear comparison between the pristine UiO-66 MOF and its Rifampicin-loaded counterpart, highlighting notable differences in surface morphology and particle arrangement. The pristine UiO-66 MOF displays a rough, textured surface composed of irregularly shaped nanoparticles with moderately uniform sizes, typically ranging from 50 to 100 nm. These particles appear slightly aggregated, which is expected for MOFs synthesized under solvothermal conditions and reflects the material’s inherent crystallinity and porous architecture as shown in Fig. 3a. In contrast, the Rif/UiO-66 MOF composite exhibits a denser, more compact structure with smoother surface features and more extensive particle fusion as indicated in Fig. 3b. This morphological transformation suggests successful drug loading, likely through surface adsorption or pore encapsulation, which results in reduced visibility of the MOF’s original porosity. The smoother texture and increased aggregation are consistent with reports on antibiotic incorporation into MOF structures, confirming the structural modifications associated with Rifampicin integration.^29^.

**Fig. 4:**
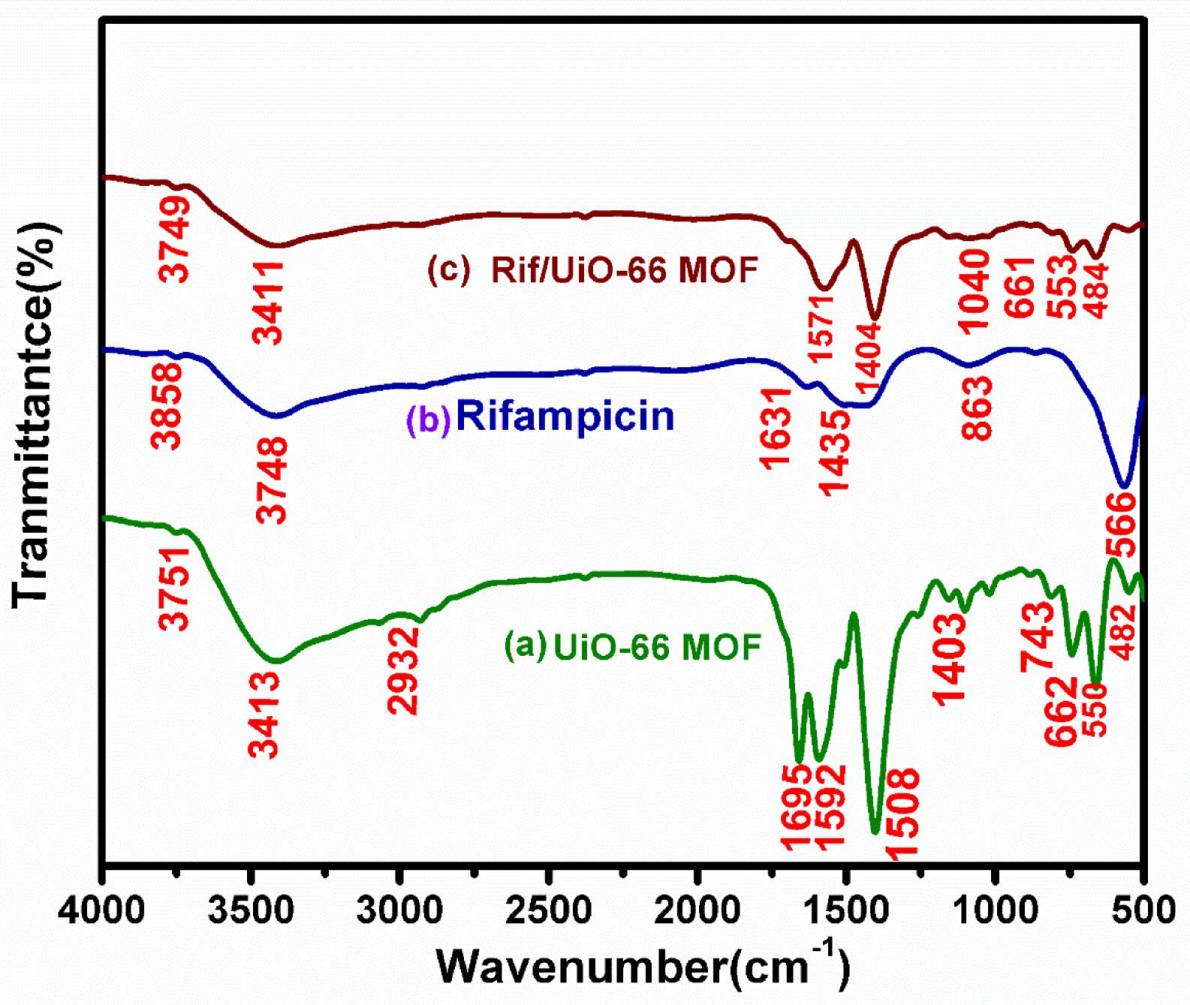
FT-IR spectra of (**a**) UiO- 66 MOF, (**b**) rifampicin, and (**c**) Rif/UiO-66 MOF.

Figure 4 illustrates the Fourier Transform Infrared (FT-IR) spectra of UiO-66 MOF, a zirconium-based metal-organic framework (MOF), reveal functional groups and bonding interactions. The 3751 cm^−1^ and 3413 cm^−1^ peaks correspond to O–H stretching vibrations, likely caused by adsorbed water molecules or hydroxyl groups on the MOF’s surface. The peak at 2932 cm⁻¹ indicates C-H stretching vibrations, possibly due to the organic linker (terephthalic acid) used in UiO-66. The peak at 1659 cm^−1^ indicates the presence of carbonyl groups, likely from the organic linker. The peaks at 1592 cm^−1^ and 1508 cm^−1^ indicate aromatic C=C stretching vibrations in the benzene ring of the organic linker. The signal at 1260 cm^−1^ is presumably caused by C–O stretching vibrations, possibly from carboxylate groups interacting with zirconium clusters. The signal at 662 cm^−1^ suggests the presence of Zr–O clusters in the UiO-66 framework. Low-frequency bands at 550 cm^−1^ and 482 cm^−1^ indicate the presence of metal-oxygen (Zr–O) stretching vibrations in MOFs. Overall, the FT-IR spectra confirm UiO-66’s successful formation, with distinct peaks corresponding to the organic linker and metal clusters.^30^.

The FT-IR spectra of rifampicin reveal functional groups that correspond to observed absorption bands. High-wavenumber peaks at 3858 cm^−1^ and 3748 cm^−1^ are likely due to O–H stretching vibrations of hydroxyl groups (–OH), indicating various types of hydroxyl groups involved in hydrogen bonding. The peak at 3414 cm^−1^ may suggest more firmly hydrogen-bonded hydroxyl groups or N–H stretching if rifampicin contains amine groups. The peak at 2926 cm^−1^ corresponds to C–H stretching vibrations, often associated with aliphatic or aromatic C–H bonds in rifampicin molecules. The peak at 1631 cm^−1^ is most likely caused by C=O stretching vibrations from rifampicin’s carbonyl groups, including amide and ketone functional groups. The 1435 cm^−1^ band may be related to C=C stretching in aromatic rings, confirming the existence of benzene rings in rifampicin’s structure. The peak at 1090 cm^−1^ may indicate C–O stretching vibrations from the ether or other oxygen-containing functional groups. The band at 863 cm^−1^ may represent C–H out-of-plane bending vibrations, reflecting the structure of the rifampicin molecule. The low-frequency peak at 566 cm^−1^ may indicate skeletal vibrations in the rifampicin molecule due to complicated ring deformations.^20^.

The FT-IR spectra of rifampicin reveal functional groups that correspond to observed absorption bands. High-wavenumber peaks at 3858 cm^−1^ and 3748 cm^−1^ are likely due to O–H stretching vibrations of hydroxyl groups (–OH), indicating various types of hydroxyl groups involved in hydrogen bonding. The peak at 3414 cm^−1^ may suggest more firmly hydrogen-bonded hydroxyl groups or N–H stretching if rifampicin contains amine groups. The peak at 2926 cm^−1^ corresponds to C–H stretching vibrations, often associated with aliphatic or aromatic C–H bonds in rifampicin molecules. The peak at 1631 cm^−1^ is most likely caused by C=O stretching vibrations from rifampicin’s carbonyl groups, including amide and ketone functional groups. The 1435 cm^−1^ band may be related to C=C stretching in aromatic rings, confirming the existence of benzene rings in rifampicin’s structure. The peak at 1090 cm^−1^ may indicate C–O stretching vibrations from the ether or other oxygen-containing functional groups. The band at 863 cm^−1^ may represent C–H out-of-plane bending vibrations, reflecting the structure of the rifampicin molecule. The low-frequency peak at 566 cm^−1^ may indicate skeletal vibrations in the rifampicin molecule due to complicated ring deformations.

**Fig. 5:**
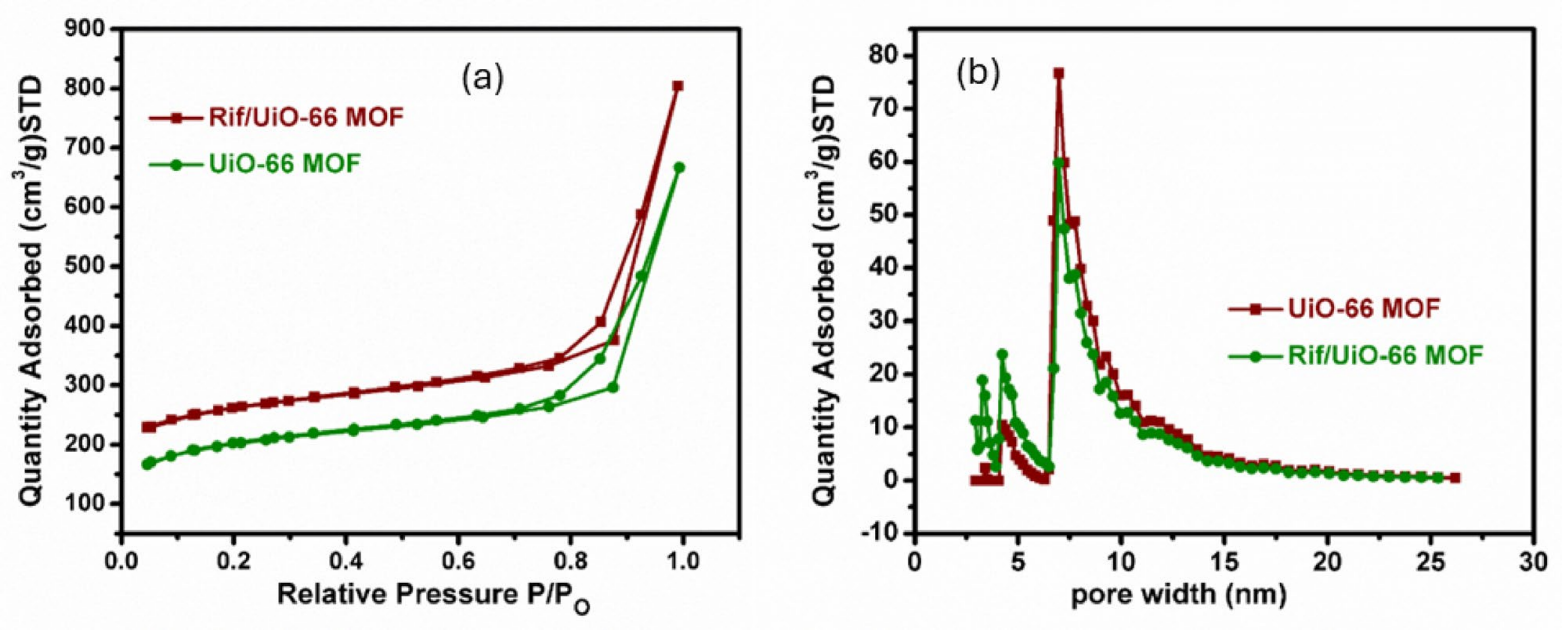
(**a**) N_2_ sorption isotherms and (**b**) pore size distribution of UiO- 66 MOF, and Rif/UiO-66 MOF.

The UiO-66 MOF and Rif/UiO-66 MOF have a mesoporous structure with wedge-shaped pores^31^, as evidenced by the Type II isotherm and H3-type hysteresis loop as shown in Fig. 5a. The average pore size distribution of around 8 nm verifies the materials’ mesoporosity as illustrated in Fig. 5b; Table 2. These materials’ mesoporous nature, combined with their well-defined pore size distribution, suggests that they could be instrumental in applications such as drug delivery, adsorption (where high surface area and mesoporosity are advantageous), and catalysis (where active sites within mesopores can increase reaction rates). These materials’ pore structure and large surface area make them ideal for applications requiring effective adsorption and regulated molecular interactions.

**Table 2:** UiO-66’s physicochemical properties:

Property	Value
Surface area (BET)	1200 m^2^/g
Pore size	8 nm
Pore volume	0.5 cm^3^/g
Thermal stability	Up to 500 °C
Crystallinity (XRD)	High
Particle size (SEM)	50–100 nm

**Fig. 6:**
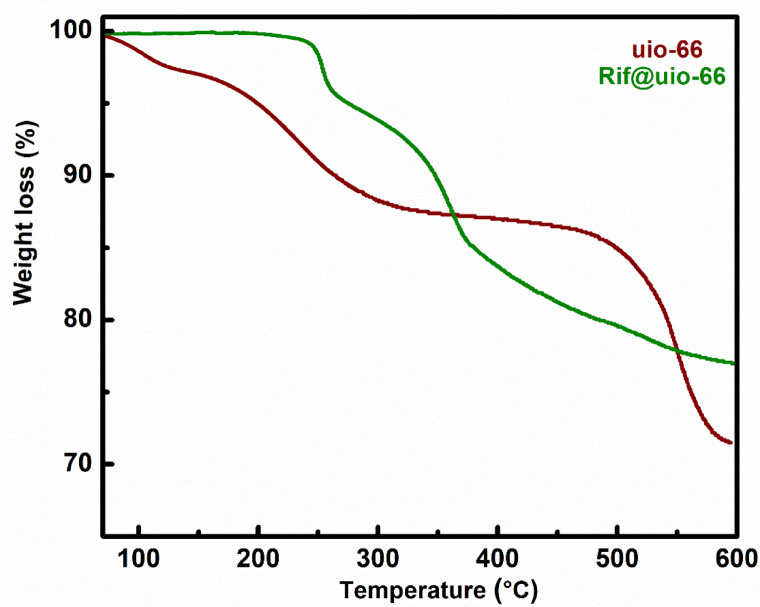
TG analysis for UiO- 66 MOF, and Rif/UiO-66 MOF.

The thermal stability of the prepared UiO-66 MOF was evaluated by thermogravimetric analysis (TGA), as presented in Fig. 6; Table 2. The UiO-66 MOF exhibits good thermal stability up to 400–500 °C, which is typical for this type of MOF due to its strong zirconium oxide clusters. The weight loss of UiO-66 MOFs begins at approximately 200 °C, primarily due to the loss of loosely bound water or solvent molecules. This initial weight loss is attributed to the absorption of moisture from the environment, which is released upon heating^32^. The weight loss increases to about 12% between 200 °C and 300 °C, indicating the loss of more tightly bound molecules, including coordinated solvents or weakly bonded organic components^32,33^. From 300 °C to 500 °C, the weight loss stabilizes around 14%, suggesting that the primary structure remains stable during this temperature range. The minimal weight change indicates that the MOF’s inorganic framework, based on zirconium oxide clusters, is resistant to decomposition.

A significant increase in weight loss begins after 500 °C, reaching approximately 27% by 600 °C. This indicates the decomposition of the MOF’s organic components, including terephthalic acid linkers. The thermal decomposition results in the collapse of both organic and inorganic parts of the MOF structure. Therefore, TGA examination indicates that UiO-66 MOF is thermally stable up to around 400–500 °C with significant degradation occurring above this temperature range due to breakdowns in its organic linkers^32,33^.

In contrast, TG analysis for Rifampicin-loaded UiO-66 (Rif/UiO-66) shows stability at around 200 °C indicating that loosely bound water or solvents have been removed before heating. At approximately 250 °C, a weight loss begins and reaches about 5%, corresponding to removal of tightly bound solvents or weakly bonded components from Rifampicin. Between temperatures ranging from roughly 250 °C and 400 °C, the weight loss increases from 5 to 15%, indicating partial decomposition of Rifampicin molecules on the MOF. This gradual nature implies that Rifampicin is breaking down at various stages unlike complex organic molecules decomposing at various stages. From approximately 400 to 500 °C, the weight loss increases to 20% and stabilizes by 600 °C. By approximately 500 °C, most decomposable organic content of the Rifampicin has been lost, while the remaining structure, likely the MOF itself, remains intact and does not further degrade. This stability reflects the robustness of the UiO-66 MOF framework even after the adsorbed drug has decomposed.

The TGA results reveal that Rifampicin adsorbed on UiO-66 MOF decomposes at 250 °C, remaining stable up to 600 °C after organic content decomposition. This information is crucial for understanding heat-related applications and ensuring drug release properties align with desired performance under varying conditions.

### Effect of adsorption operational parameters

#### Effect of pH

**Fig. 7:**
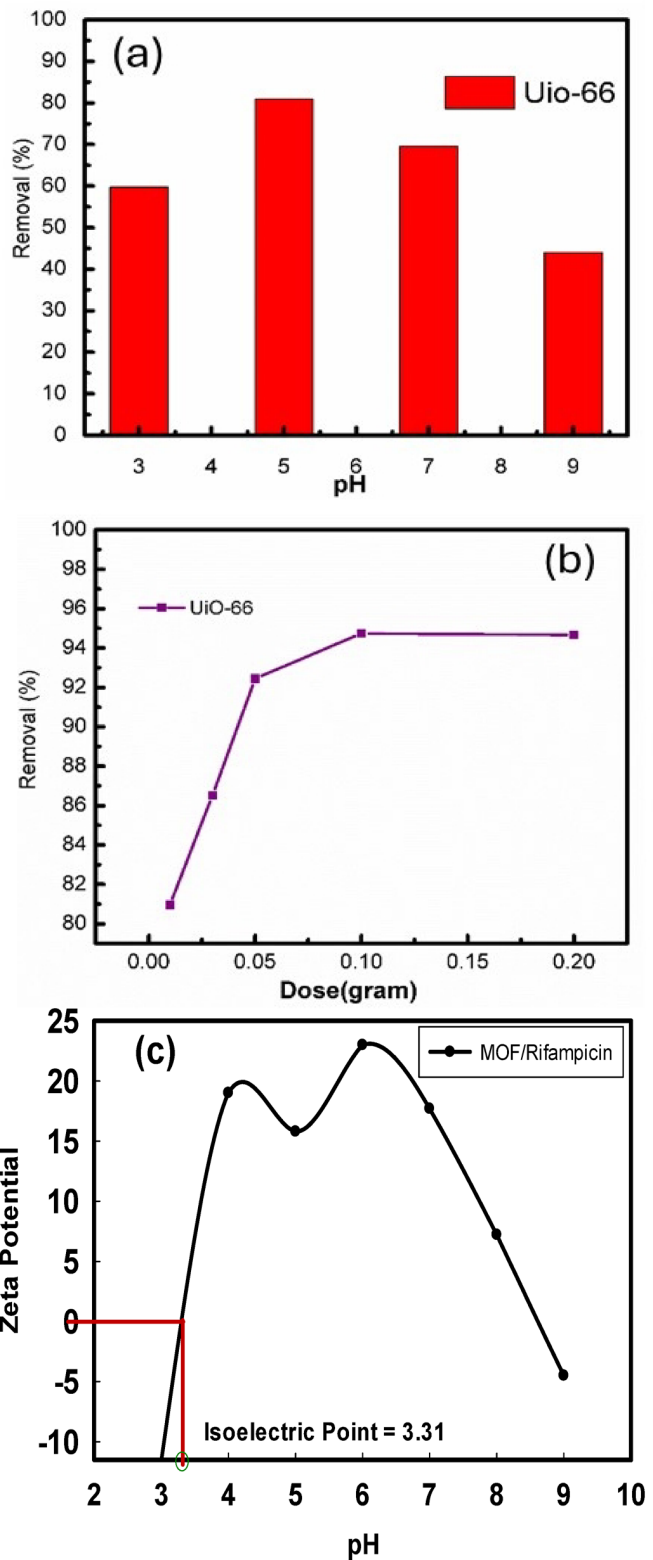
Effect of (**a**) different pH, and (**b**) adsorbent dose on the adsorption of rifampicin onto UiO-66, and (**c**) zeta potential of MOF/rifampicin composite at different pH.

The efficiency of UiO-66 in eliminating rifampicin varies according to the pH of the solution. At pH 3, the removal efficiency is 59.74%, indicating that adsorption is moderate. When the pH is adjusted to 5, the removal efficiency increases significantly to 80.96%, demonstrating that this pH is optimal for adsorption. However, when the pH is neutral (7), the efficiency reduces to 69.57%, indicating a decline in adsorption performance. At pH 9, removal effectiveness drops to 43.65%, making it the least effective adsorption condition as shown in Fig. 7a.

These findings indicate that UiO-66 MOF works best at a slightly acidic pH of 5 to remove rifampicin. Lower adsorption effectiveness is seen under both more acidic (pH 3) and more basic conditions (pH 7 and 9). The interpretation is that UiO-66 MOF, like many metal-organic frameworks (MOFs), has a surface charge that varies with pH. At lower pH values (acidic conditions), UiO-66’s surface is likely to be positively charged, whereas at higher pH values (basic conditions), it becomes more negatively charged. On the other hand, rifampicin is a zwitterionic molecule, which, means it may carry both positive and negative charges depending on the pH. At a somewhat acidic pH of 5, rifampicin’s charge distribution may encourage strong interactions with the surface of UiO-66 MOF, hence increasing adsorption. The ideal pH = 5 appears to be the point at which the surface charge of UiO-66 MOF and rifampicin’s ionization state are best aligned for maximal adsorption efficiency. This could be owing to a combination of electrostatic attraction and other interactions (e.g., hydrogen bonding, van der Waals forces) that work best at this pH. At a lower pH (pH 3), adsorption is less efficient, potentially due to higher competition from hydrogen ions or a lower charge interaction. However, at higher pH levels (pH 7 and pH 9), the drop in adsorption efficacy could be attributed to repulsion between similarly charged surfaces or changes in rifampicin’s ionization state, making it less compatible with the UIO-66 MOF surface.

#### Effect of UiO-66 MOF dose

Figure 7b. shows that, as the dose of UiO-66 MOF increases from 0.01 g to 0.05 g, the removal efficiency of rifampicin rises from 80.9 to 94.7%. This suggests that greater dosages of UiO-66 MOF improve adsorption, most likely due to increased active sites where rifampicin molecules can bind. The removal efficiency plateaus at 94.7% for dosages of 0.03 g and above, indicating that a saturation threshold has been achieved. At this time, the majority of the available rifampicin molecules have been adsorbed onto the UiO-66 MOF, and adding more adsorbent does not significantly improve removal efficiency. This implies that, while increasing the dose initially has a considerable impact, adding more UiO-66 MOF after a certain point does not result in greater rifampicin removal. The optimal dose for efficient elimination is around 0.1 gm since raising it further does not result in any apparent improvement.

#### Zeta potential and isoelectric point of mof/rifampicin composite

Figure 7c shows the zeta potential profile of the MOF/rifampicin composite as a function of pH provides insight into its surface charge behavior and stability in suspension. The isoelectric point (IEP), defined as the pH where the zeta potential is zero and the surface charge is neutral, is observed at approximately pH 3.32. At this point, the net repulsive forces are minimized, increasing the tendency for particle aggregation. Below this pH, the surface acquires a net positive charge, while above it, the surface becomes increasingly negative. The composite shows enhanced electrostatic interaction and adsorption efficiency at pH 5, where the zeta potential is strongly positive (around + 22 mV), suggesting favorable conditions for binding negatively charged species like Rifampicin. In contrast, the zeta potential decreases beyond pH 6 and reaches negative values (around − 7 mV at pH 9), indicating lower surface charge density and reduced colloidal stability. The data demonstrate that maximum stability and adsorption performance occur in slightly acidic conditions, with reduced stability near the IEP due to decreased electrostatic repulsion. This behavior aligns with established colloidal science, which states that higher absolute zeta potential values (± 30mV) indicate better dispersion stability, while values near zero promote flocculation^34^.

#### Effect of rifampicin concentration on adsorption by UiO-66

**Fig. 8:**
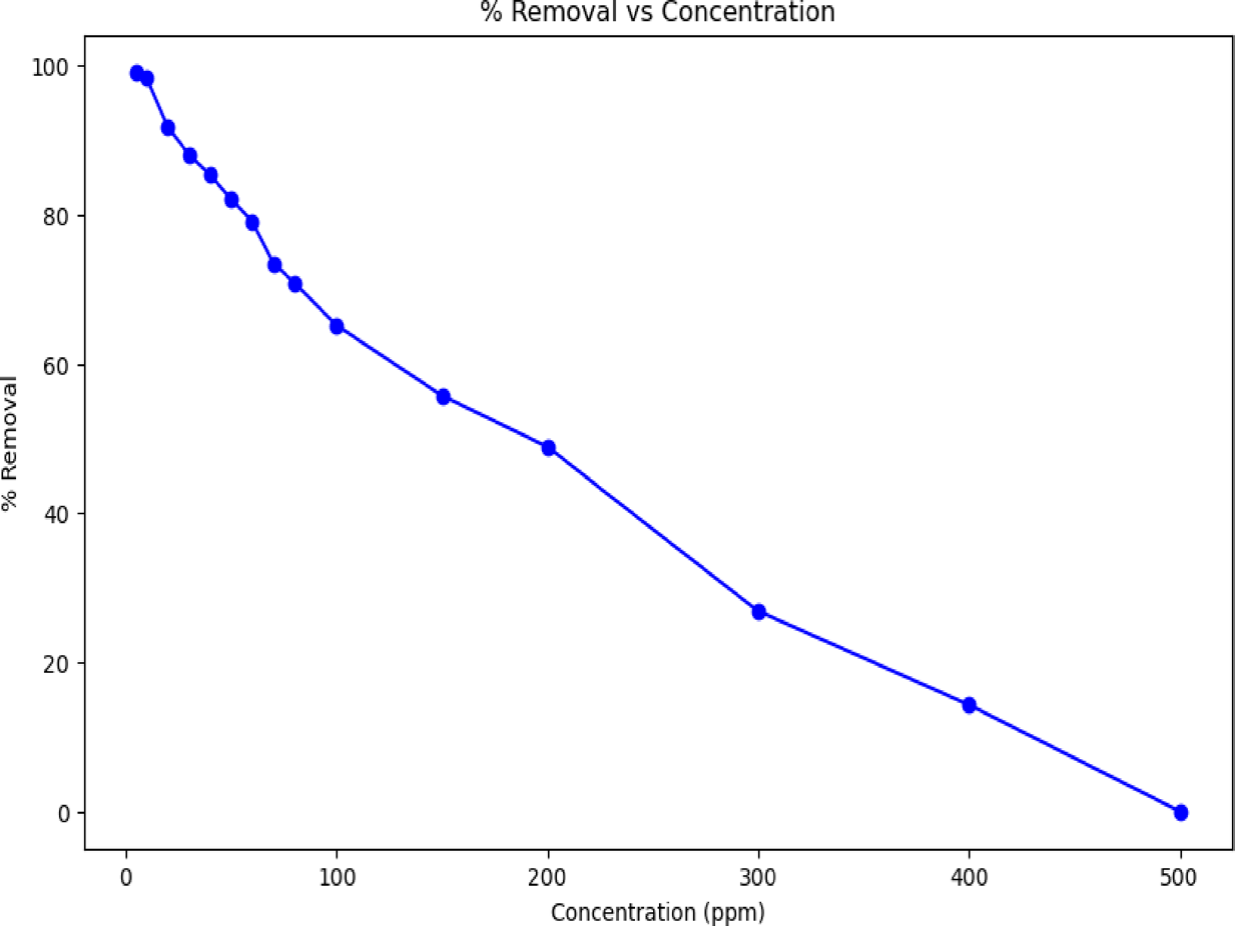
Effect of rifampicin concentration on adsorption by UiO-66.

The adsorption efficiency of UiO-66 metal-organic frameworks (MOFs) for rifampicin is significantly influenced by the initial concentration of the antibiotic. Figure 8 indicated that at lower concentrations (10–50 ppm), UiO-66 exhibits high removal efficiencies, ranging from 98.34 to 82.09%, due to the abundance of available adsorption sites. However, as the concentration increases (60–100 ppm), the removal efficiency gradually decreases from 79.09 to 65.14%, indicating the progressive saturation of active sites on the MOF surface. At higher concentrations (150–500 ppm), a more pronounced decline in removal efficiency is observed, dropping to as low as 0.00%, which suggests that the adsorption capacity of UiO-66 is exceeded, leading to site saturation and reduced performance. This trend aligns with findings from studies on similar MOFs, where increased pollutant concentrations result in decreased adsorption efficiency due to limited active sites. Therefore, optimizing the initial concentration of rifampicin is crucial for maximizing the adsorption efficiency of UiO-66 in wastewater treatment applications.

### Adsorption investigations (kinetics, isotherms)

**Fig. 9:**
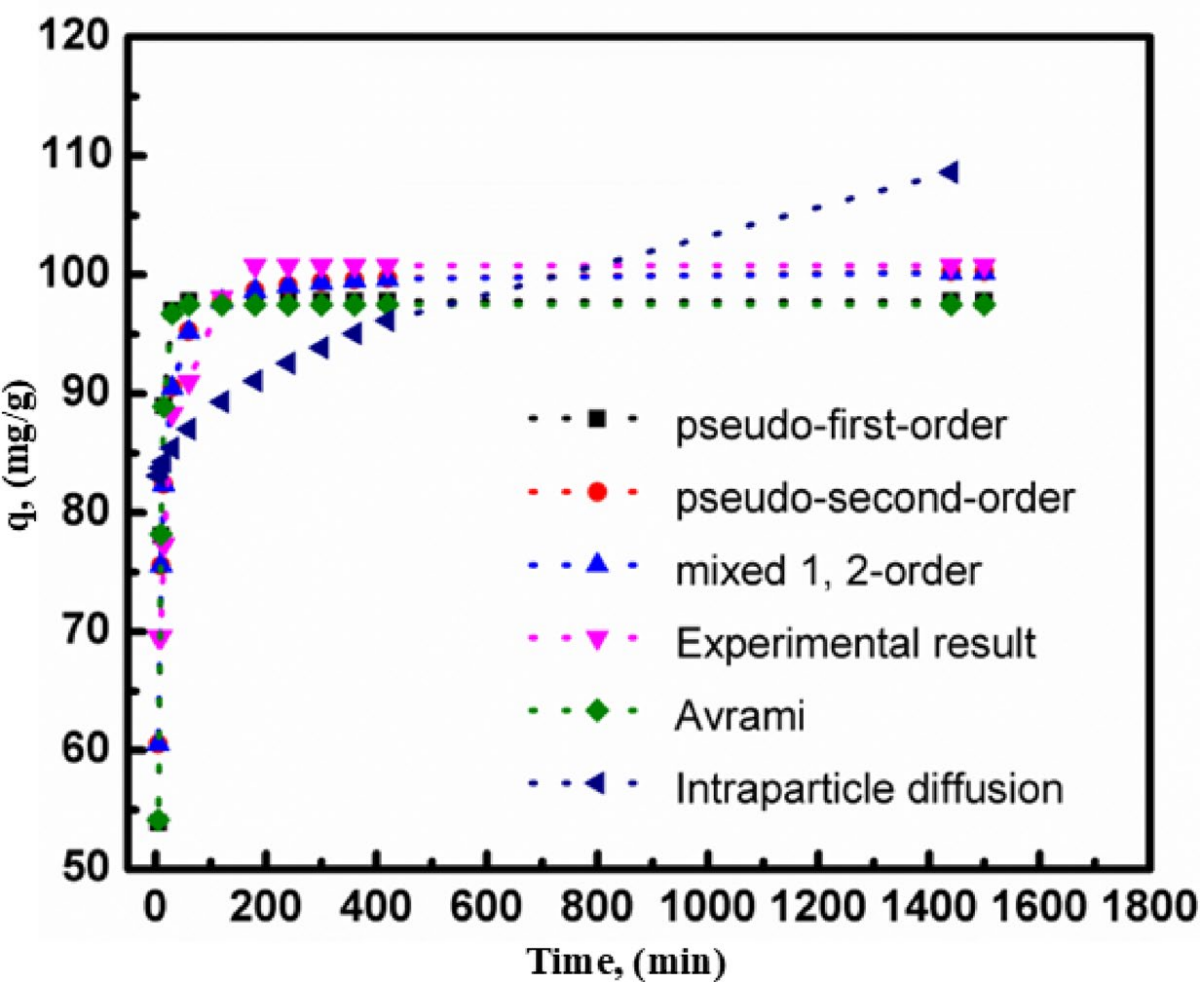
Kinetic parameters of UiO-66 prepared for the removal of rifampicin from wastewater.

Adsorption kinetics studies have examined the effect of contact time on the adsorption process over a range of 0–105 min utilizing several models, such as pseudo-first-order^35^, second-order, mixed order^36^, the Avrami model^37^, and intraparticle diffusion. Figure 9 illustrates the sharp increase in removal efficiency for rifampicin during the first 180 min. This is because longer adsorption times increase the likelihood of an adsorbent-pollutant interaction. The removal efficiency vs. time graph peaked after 180 min, indicating that the system had reached equilibrium. The parameters of the kinetic model are shown in Table 3; Fig. 9. According to the experimental findings, rifampicin could bind to UiO-66 MOF^13^. The experimental results showed that the adsorption of rifampicin on the prepared UIO-66 MOF adsorbent followed a pseudo-second-order and mixed 1, 2-order model with an R^2^ of 0.98. The pseudo-second-order model implies that the rate-limiting phase is chemisorption, which involves valence forces caused by electron sharing or exchange between the adsorbent and the adsorbate. It is commonly used when adsorption is thought to be governed by chemical interactions, such as hydrogen bonding or coordination with active sites on UiO-66 rather than purely physical processes, likely through van der Waals forces or surface diffusion. Suppose the experimental data agree well with this model. In that case, it implies that the adsorption capacity is directly proportional to the number of active sites available on UiO-66 MOF and that the process is more likely to be chemisorption than physisorption. While, the mixed 1,2-order model considers both first- and second-order kinetics, indicating that multiple mechanisms might control the process simultaneously. The first phase may be dominated by physical adsorption, with rapid uptake due to surface attraction. As the process develops, chemical adsorption becomes more important, resulting in higher binding forces and possibly the creation of a monolayer on the adsorbent surface. This model can be beneficial when the adsorption process does not precisely adhere to a single kinetic order but involves several distinct mechanisms.

**Table 3:** Kinetic parameters of UiO-66 prepared for the removal of rifampicin from wastewater.

Model	Equation	Parameter	Value	*R* ^2^
Pseudo first order	$\:\text{ln}\left({q}_{e}-{q}_{t}\right)=\text{l}\text{n}\:{q}_{e\:}-\:{\text{k}}_{1\:}t$	q_e cal_	97.795	
		K_1_	0.160	0.94
Pseudo second order	$\:\frac{\text{t}}{{\text{q}}_{\text{t}}}=\frac{1}{{\text{k}}_{2}{\text{q}}_{\text{e}}^{2}}+\frac{\text{t}}{{\text{q}}_{\text{e}}}$	q_e cal_	100.48	
		K_2_	0.003	0.98
Intraparticle diffusion model	$\:{q}_{t}\equiv\:{\text{k}}_{\text{i}\text{p}\:}\sqrt{\text{t}}+{\text{C}}_{\text{i}\text{p}}$	C_ip_	79.68	
		K_ip_	0.9	0.95
Avrami model	$\:\:\text{ln\:}\{\text{l}\text{n}\:\left[{\text{q}}_{\text{e}}/\left({\text{q}}_{\text{e}}-{\text{q}}_{\text{t}}\right)\right]\}\:\:\:\:=\text{n}\:\text{l}\text{n}\:\text{k}+\text{n\:ln}\text{t}$	q_e_	97.08	
		K_av_	0.416	0.94
		N_av_	0.39	
The Mixed 1,2-order model	q_t_ = q_e_ *(1-exp(-kt)* 1-f_2_ exp(-kt)	K	0.001	0.98
		q_e_	100.26	
		f_2_	0.996	

**Fig. 10:**
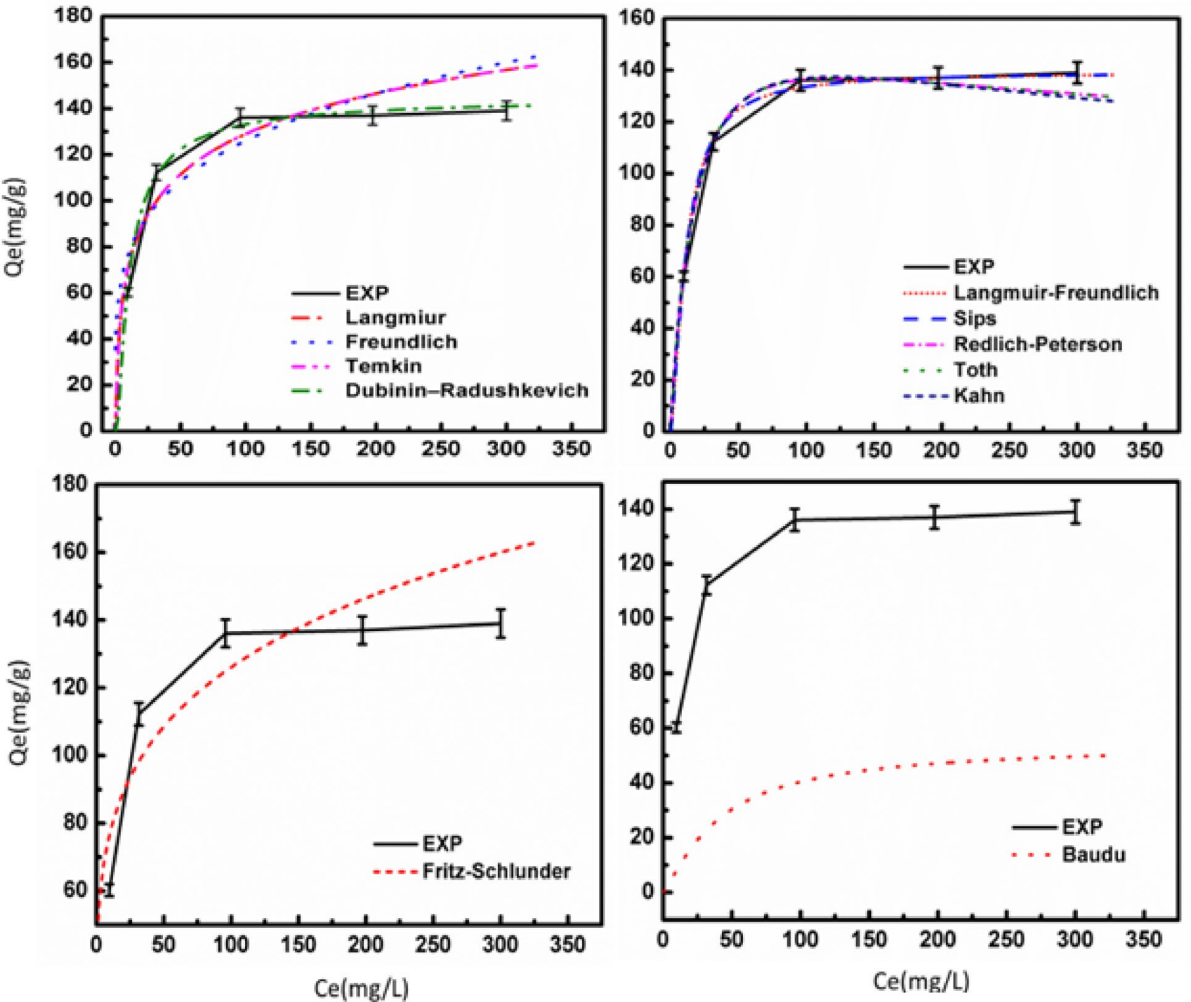
Isotherm parameters of UiO-66 MOF prepared for the removal of rifampicin from wastewater.

Adsorption isotherm models were used to investigate the adsorption capabilities of the generated adsorbent. Eleven nonlinear equilibrium isotherm models were utilized to fit the rifampicin experimental data as shown in Fig. 10. The Langmuir, Freundlich, Temkin, and Dubinin–Radushkevich isotherm models (with two parameters) were utilized. The Langmuir–Freundlich, Sips, Redlich–Peterson, Toth, and Kahn isotherm models, all of which have three parameters, were employed. Two research used the higher-parameter isotherms Baudu and Fritz Schlunder^38^, as shown in Table 4. Figure 10 depicts the isotherm models for rifampicin adsorption on the generated UiO-66. Rifampicin had greater q_max_ and R^2^ values when using the Fritz–Schlunder models. The Fritz–Schlunder isotherm is useful for analyzing complex adsorption patterns, such as those of rifampicin on UiO-66 MOF due to their heterogeneous surface and varying pore sizes. This model can account for the preferential adsorption of rifampicin molecules onto specific sites or changes in the adsorption capacity at different concentrations. The Fritz–Schlunder isotherm can also accurately model multi-layer adsorption or cooperative effects, where one molecule’s adsorption influences another’s, making it more accurate than simpler isotherms. By adjusting exponents, the isotherm can accurately reflect the adsorption behavior of rifampicin on UiO-66 MOF. These results confirmed the kinetics statistics, as previously reported^36^.

**Table 4:** Isotherm parameters of UiO-66 prepared for the removal of rifampicin from wastewater.

Model	Equation	Parameter	Value	Q _max_	*R* ^2^
Two parameters					
Langmuir isotherm	$\:{q}_{e}={q}_{{max}}\left(\frac{{k}_{L}{C}_{e}}{1+{k}_{L}{C}_{e}}\right)$	K_L_	44,932	110.88	0.97
Freundlich isotherm	$$\:{q}_{e}={k}_{F}\:({{C}_{e})}^{\raisebox{1ex}{$1$}\!\left/\:\!\raisebox{-1ex}{$n$}\right.}$$	1/n	0.217	–	0.995
		K_F_	46.38	–	0.997
Temkin isotherm	$\:{q}_{e}=\frac{RT}{{A}_{T}}{ln}{A}_{T}+\frac{RT}{{A}_{T}}Ln\:{C}_{e}$	RT	8.31		
		A_T_	1.64		
Dubinin‒Radushkevich isotherm	$\:{q}_{e}=\left({q}_{max}\right){exp}\left(-{k}_{ad}{\epsilon\:}^{2}\right)$	K_ad_	0.0017	145.19	0.9997
Three parameters isotherm					
Langmuir–Freundlich isotherm	$\:{q}_{e}=\frac{{q}_{max}{\left({k}_{LF}\:{C}_{e}\right)}^{\text{M}\text{L}\text{F}}}{1+{\left({k}_{LF\:}{C}_{e}\right)}^{\text{M}\text{L}\text{F}}}$	K_LF_	0.086	139.188	0.999
		M_LF_	1.47		
Sips isotherm	$\:{q}_{e}=\frac{{\left({q}_{max}{k}_{s}\:{C}_{e}\right)}^{1/n}}{{\left(1+{k}_{s}\:{C}_{e}\right)}^{1/n}}$	K_S_	0.027	139.189	0.999
		1/n	1.47		
Redlich–Peterson isotherm	$\:{q}_{e}=\frac{{k}_{R\:\:}{C}_{e}}{1+{a}_{R\:\:}{{C}_{e}}^{\beta}}$	K_R_	8.5	–	1
		a_R_	0.026		
		β	1.149		
Toth isotherm	$\:{q}_{e}=\frac{{k}_{e}\:{C}_{e}}{{\left(1+{\left({k}_{L}\:{C}_{e}\right)}^{n}\right)}^{1∕n}}$	K_e_	9.76	–	1
		K_L_	0.026		
		N	1.15		
Kahn isotherm	$\:{q}_{e}=\frac{{q}_{max}{\:b}_{k\:}{C}_{e}}{{\left(1+{b}_{k\:}{C}_{e}\right)}^{{a}_{k}}}$	b_k_	0.037	243.58	0.9999
		a_k_	1.218		
Higher parameter isotherm					
Baudu isotherm	$\:{q}_{e}=\frac{{q}_{max}{\:b}_{0}{\:{C}_{e}}^{1+X+Y}}{1+{b}_{0}{\:{C}_{e}}^{1+X}}$	b_o_	0.01	49.9	0.998
		X	0.012		
		Y	0.248		
Fritz–Schlunder isotherm	$\:{q}_{e}=\frac{{q}_{{max}_{Fss}}{k}_{1}{{C}_{e}}^{{m}_{1}}}{1+{k}_{2}{{C}_{e}}^{{m}_{2}}}$	K_1_	0.855	54.2	0.995
		K_2_	0		
		m_1_	0.217		
		m_2_	0		

### Adsorption mechanism

**Fig. 11:**
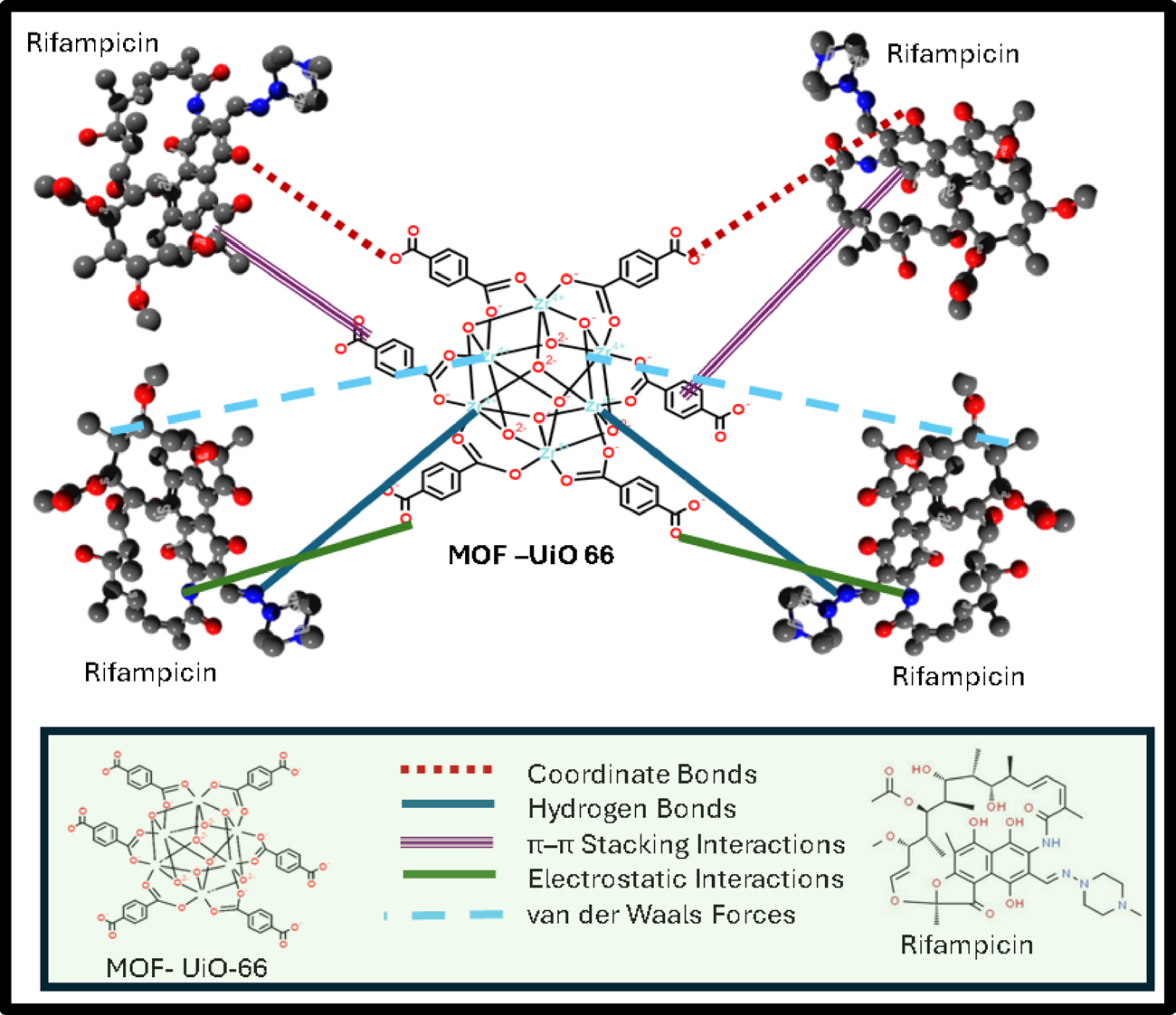
Adsorption mechanism for the rifampicin onto UiO-66 MOF.

The interaction between rifampicin and the UiO-66 metal–organic framework was explored through a combination of structural, spectroscopic, thermal, and adsorption studies^13^. X-ray diffraction (XRD) analysis revealed that the MOF maintained its crystalline structure after drug loading, as the key diffraction peaks remained unchanged. These consistent peaks, particularly those observed at specific 2θ values such as 7.14°, 8.56°, and 25.42°, confirm that the framework retains its stability^39^, which is essential for preserving its porosity and adsorption capacity^40^. The incorporation of rifampicin into the UiO-66 metal–organic framework (MOF) results in notable shifts in Fourier-transform infrared (FT-IR) spectra, indicating the formation of various interactions that enhance the adsorption process^41,42^. Hydrogen bonding is a primary interaction, where hydroxyl and amine groups of rifampicin form bonds with the oxygen atoms of the MOF’s carboxylate groups, leading to shifts in the O–H stretching vibrations in the FT-IR spectra. Coordinate bonds also play a significant role; the nitrogen atoms in rifampicin can donate electron pairs to the zirconium centers in UiO-66, forming stable complexes that are reflected in the FT-IR spectra by shifts in the C=N stretching vibrations. π-π interactions occur between the aromatic rings of rifampicin and the organic linkers of the MOF, contributing to the overall stability of the complex. Van der Waals forces, though weaker, facilitate the initial attraction and stabilization of rifampicin within the MOF’s pores. Electrostatic attractions between charged groups of rifampicin and the MOF further stabilize the drug within the framework. Collectively, these interactions, evidenced by specific shifts and intensity changes in the FT-IR spectra, confirm the successful incorporation of rifampicin into UiO-66 and highlight the multifaceted nature of the adsorption process. In terms of surface characteristics, BET analysis confirmed that UiO-66 possesses a large surface area and mesoporous structure, with an average pore size near 8 nm. The observed Type II isotherm and H_3_ hysteresis loop point to the presence of slit-like pores that can effectively accommodate drug molecules^40^. Morphological examination using scanning electron microscopy (SEM) showed relatively uniform, spherical particles with minor agglomeration, offering a broad surface area for drug contact and diffusion. Thermal stability, assessed through thermogravimetric analysis (TGA), indicated that UiO-66 remains stable up to around 500 °C. A gradual weight loss began at approximately 200 °C due to the release of absorbed solvents, while the main framework decomposition occurred above 500 °C. In the rifampicin-loaded composite, the onset of weight loss was slightly shifted, starting at 250 °C, which corresponds to the breakdown of the drug within the pores, while the MOF structure itself remained thermally intact^40^. To better understand the adsorption behavior, kinetic studies were performed and found to fit a pseudo-second-order model, suggesting that chemical interactions such as electron sharing or exchange dominate the adsorption process. This model also reflects that the rate of adsorption is influenced by the availability of active binding sites on the MOF surface. Finally, equilibrium adsorption data were analyzed using Langmuir and Freundlich isotherm models. The results fit well with the Langmuir model, indicating monolayer adsorption on a uniform surface^43^. The high value of maximum adsorption capacity (q_max_) obtained supports the strong affinity of UiO-66 for rifampicin, confirming its potential as an efficient drug carrier^13^ as shown in Fig. 11.

### Recyclability of UiO-66 MOF

**Fig. 12:**
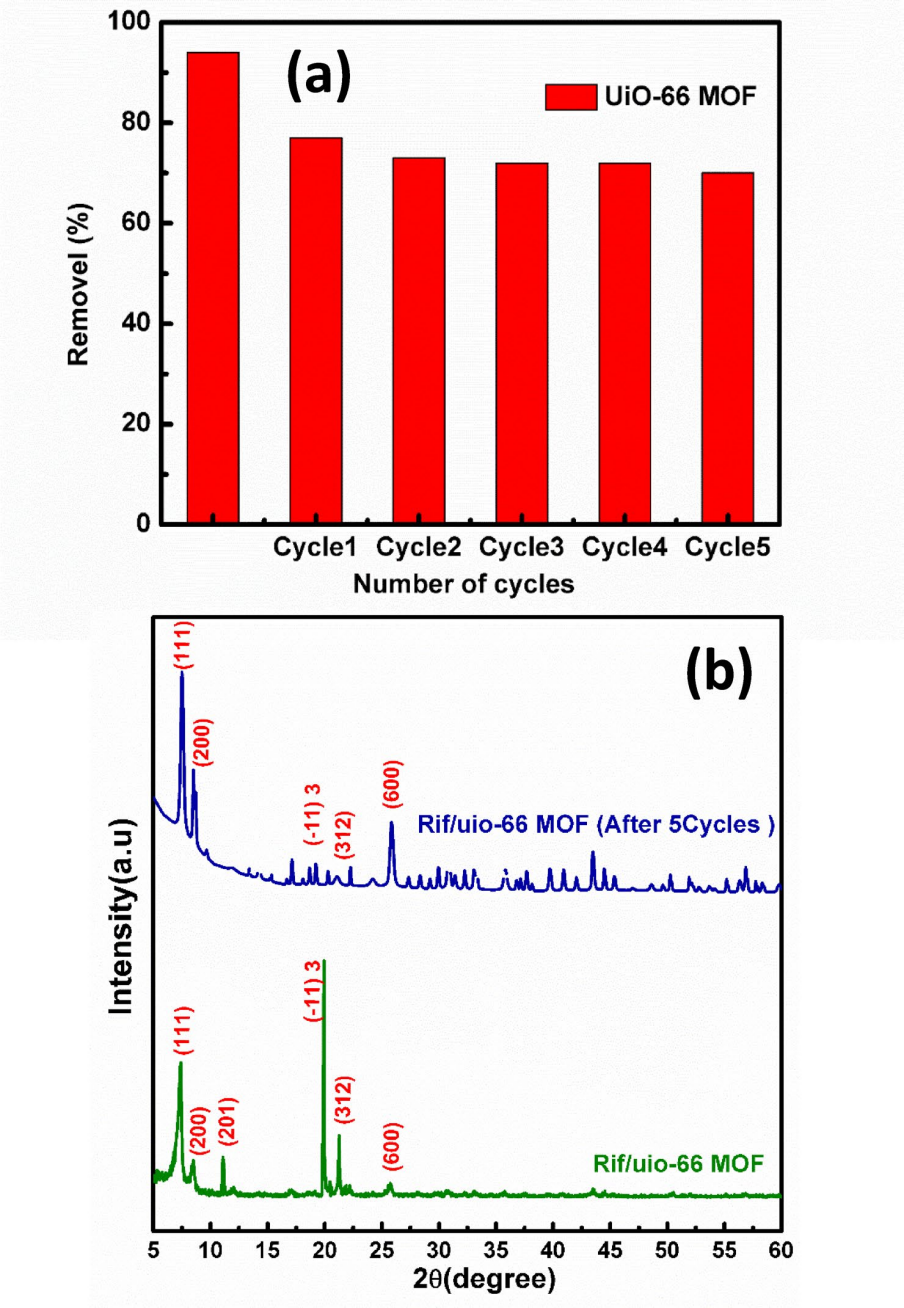
(**a**) Regeneration and reuse tests for the adsorption of rifampicin onto UiO-66 MOF, (**b**) X-ray diffraction analysis of the Rif/UiO-66 MOF composite after five cycles.

The UiO-66 MOF has been observed to show a gradual decrease in removal efficiency across multiple cycles, suggesting a reduction in the adsorptive capacity as shown in Fig. 12a. This reduction can be attributed to several factors, including partial pore blockage or structural degradation, incomplete rifampicin desorption, and adsorbate-induced surface chemistry changes. Ethanol, a commonly used solvent for desorption, has mixed effects on the MOF’s effectiveness as a desorption agent and structural integrity^44^. While ethanol is relatively effective in removing adsorbed rifampicin, its efficiency seems to decline with each cycle due to its inability to access deeply adsorbed molecules or desorb rifampicin that has formed strong interactions with the MOF surface. Solvent-induced degradation may also cause slight swelling or structural changes to the UiO-66 framework, affecting its structural stability and adsorptive capacity^6^. Despite the decline in efficiency, the MOF retains a reasonable adsorption capacity after five cycles (70%), making it a cost-effective and viable option for applications where some loss of efficiency is acceptable over time. To enhance recyclability, strategies to improve recyclability include exploring alternative desorption protocols, functionalizing or coating UiO-66 MOF, and optimizing regeneration techniques. By incorporating hydrophilic or hydrophobic groups into the MOF’s surface chemistry, modifying the surface chemistry may enhance both adsorption capacity and desorption efficiency, reducing the rate of decline in performance over multiple cycles. Additionally, optimizing regeneration techniques such as thermal treatment or using supercritical fluids could potentially restore the MOF’s structure and adsorption capacity more effectively than ethanol, minimizing the loss of efficiency over repeated cycles^44^.

Figure 12b shows the X-ray diffraction (XRD) analysis of the Rif/UiO-66 MOF composite after five adsorption–desorption cycles reveal important insights into its structural stability and performance. The diffraction pattern shows that the primary characteristic peaks of UiO-66 such as those at 2θ ≈ 7.1° (111), 8.5° (200), 11.9° (201), 14.4° (− 113), 17.3° (312), and 25.4° (600) are retained after multiple cycles, indicating that the MOF largely preserves its crystalline framework. However, the cycled material exhibits a noticeable reduction in peak intensity, peak broadening, and the appearance of additional minor peaks, suggesting partial loss of crystallinity, structural disorder, and possible adsorbate-induced modifications^45^. These changes are likely due to factors such as incomplete rifampicin desorption, pore blockage, and repeated exposure to ethanol, which, while initially effective, becomes less efficient in removing deeply adsorbed molecules over successive cycles and may induce structural swelling or chemical changes on the MOF surface. Despite these alterations, the core structure of the MOF remains intact, maintaining around 70% of its adsorption efficiency, which supports its continued viability for drug delivery or pollutant removal applications^46^. The retained diffraction peaks of rifampicin within the composite also suggest that the drug remains structurally stable within the MOF, further affirming the robustness of the composite. To mitigate the observed decline in performance, alternative desorption solvents, surface functionalization of the MOF, or improved regeneration strategies such as thermal or supercritical fluid treatments should be explored. These enhancements may help restore the structural integrity and improve the recyclability of the MOF for extended use^47,48^.

### MTT assay

**Fig. 13:**
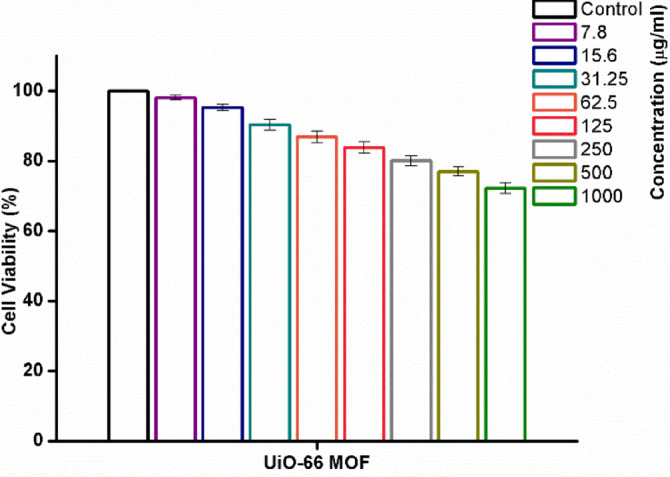
The effect of UiO-66 on cell viability of HL-7702 cell line. Cells were treated with UiO-66 at various concentrations (0–1000 µg/ml) for 24 h. The results represent the means of three separate experiments, and the error bars represent the standard error of the mean.

The study on UiO-66 nanoparticles and their effect on HL-7702 liver cells reveals a concentration-dependent cytotoxicity, as shown in Fig. 13. At lower concentrations (7.8 to 62.5 µg/ml), cell viability remains near 100%, indicating minimal cytotoxic effects and good biocompatibility. As the concentration increases to 125 µg/ml, a slight decrease in viability is observed, with more pronounced reductions at 250 µg/ml (approximately 80.12% viability) and 500 µg/ml (about 77% viability). At the highest concentration of 1000 µg/ml, cell viability is around 72%. These findings suggest that while UiO-66 nanoparticles are generally safe at low concentrations, higher doses can lead to considerable cytotoxic effects, highlighting the importance of careful dosing in potential biomedical and water treatment applications.

### Antimicrobial study

#### Determination of MIC and MBC

**Fig. 14:**
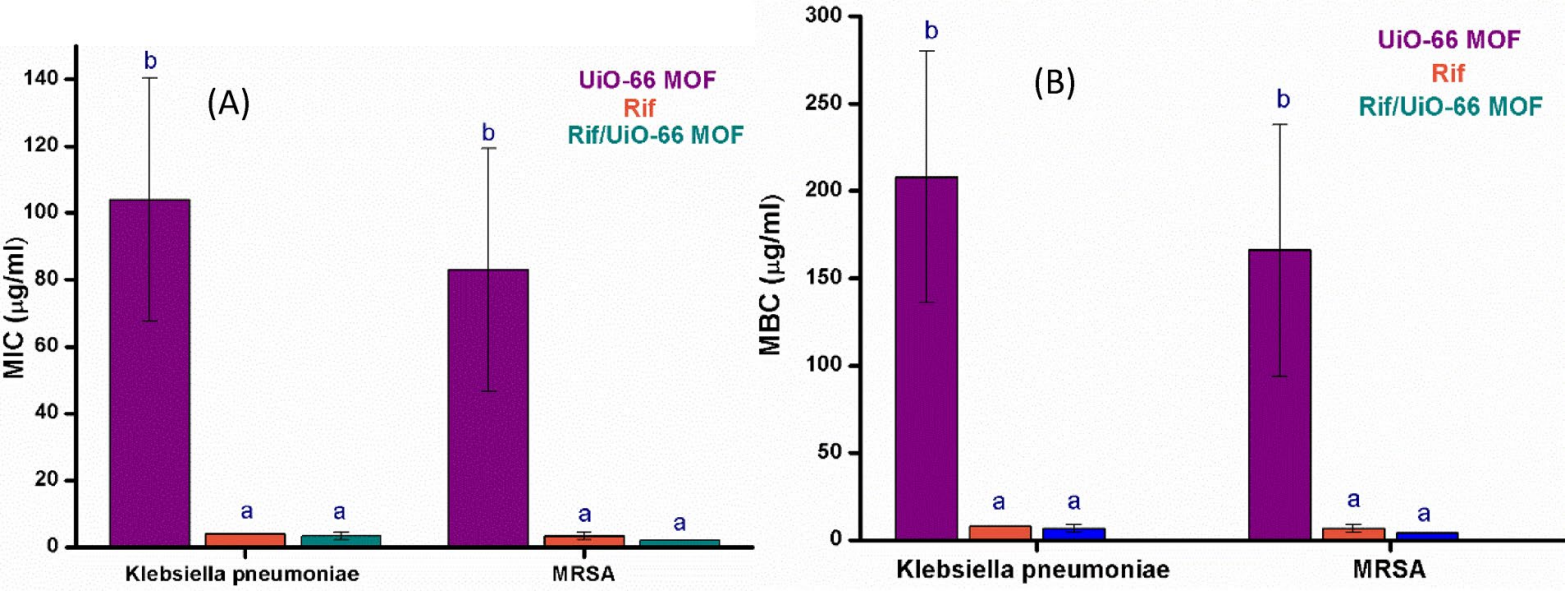
(**A**) MIC and (**B**) MBC values for UiO-66 MOF, Rifampicin and Rif/ UiO-66 MOF against K. pneumoniae and MRSA. Columns bearing the same letter indicate no significant difference at *p* < 0.05 while different letters indicating significant difference at *p* < 0.05.

As illustrated in Fig. 14, the results for MIC and MBC of UiO-66 MOF nanoparticles, Rif, and Rif/UiO-66 MOF nanoparticles against Klebsiella pneumoniae and MRSA reveal distinct differences in antibacterial efficacy. The UiO-66 MOF nanoparticles alone require significantly higher concentrations to achieve both MIC and MBC, indicating limited antibacterial activity compared to rifampicin and the Rif/UiO-66 MOF combination. For both bacterial strains, rifampicin and the Rif/UiO-66 MOF combination show much lower MIC and MBC values, suggesting enhanced effectiveness. The combination of Rif/UiO-66 MOF demonstrates a potential synergistic effect, achieving slightly lower concentrations than rifampicin alone, which may improve antibiotic delivery or potency. Statistically significant differences are indicated by different letters, with UiO-66 MOF consistently showing higher values (marked ‘b’) than rifampicin and Rif/UiO-66 MOF (marked ‘a’). This suggests that while UiO-66 MOF has some inherent antibacterial properties, its combination with rifampicin offers a more potent treatment option against these pathogens. The pH of the surrounding environment plays a crucial role in determining the effectiveness of antimicrobial nanomaterials, including MOF-based drug delivery systems.

The pH of the microenvironment can significantly influence the efficacy of antimicrobial nanomaterials, including MOF-based drug delivery systems. Several factors contribute to this pH-dependent behavior. Firstly, the structural integrity of the MOF carrier itself is crucial. UiO-66 is recognized for its notable chemical stability, particularly in aqueous media across a relatively wide pH range, often reported to be stable from acidic conditions (pH = 1–3) up to neutral or mildly alkaline conditions (pH = 8–9)^49–51^. However, exposure to extreme pH values, especially highly alkaline environments (pH > 9–10), or specific buffer components can lead to degradation^51–53^, potentially causing uncontrolled drug release and loss of the nanocarrier structure. Secondly, the release kinetics of the encapsulated drug, Rifampicin in this case, from the UiO-66 pores may be pH-sensitive. Variations in pH can alter the protonation state of both the drug molecule and the MOF’s internal surface functional groups, thereby affecting drug-framework interactions (e.g., hydrogen bonding, electrostatic forces) and modulating the release rate^54^. Thirdly, the surface charge of the MOF particles, governed by the environmental pH relative to the material’s isoelectric point (IEP), plays a critical role in interactions with bacterial cells. Bacterial surfaces typically carry a net negative charge at physiological pH [7]. Consequently, at pH values below the IEP, the MOF particles would exhibit a positive surface charge, potentially enhancing electrostatic attraction to bacteria and facilitating adhesion or uptake. Conversely, at pH values above the IEP, a negative surface charge on the MOF could lead to electrostatic repulsion, potentially hindering interaction^55^. While the specific IEP of the Rif/UiO-66 composite was not determined in this study, understanding these charge dynamics is key to predicting pH-dependent interactions. Finally, it is important to consider that pH itself directly impacts bacterial viability and growth rates [9]. Therefore, evaluating the antimicrobial activity across different pH values requires careful consideration of MOF stability, pH-triggered drug release, surface charge interactions, and the direct effects of pH on the target microorganisms.

## Conclusion

The study showcases the potential of UiO-66, a zirconium-based MOF, for adsorption of rifampicin and enhanced antibacterial activity. The MOF demonstrated a high adsorption capacity of 542 mg/g under optimized conditions, confirming its effectiveness in adsorbing antibiotic contaminants from wastewater, offering an affordable and sustainable solution for environmental remediation. UiO-66 nanoparticles are biocompatible at low concentrations but exhibit notable cytotoxic effects at higher levels, emphasizing the need for careful dosing in biomedical applications. The combination of Rif/UiO-66 MOF demonstrates superior antibacterial effectiveness, suggesting a promising synergistic approach for treating infections caused by K. pneumoniae and MRSA. UiO-66 MOF, a dual-functional adsorbent and antimicrobial agent, has potential applications beyond wastewater treatment. It can effectively target and neutralize bacterial infections. Future research should focus on experimentally determining the isoelectric point of the Rif/UiO-66 composite, and antimicrobial efficacy to fully elucidate its behavior in diverse physiological environments optimizing its properties, scaling its use in industrial settings, and investigating its interactions with other drugs to enhance its multifunctional capabilities.

## Data Availability

We have now included a Data Availability Statement in the manuscript under the Declaration section as follows: “The datasets generated and analyzed during the current study are available from the corresponding author upon reasonable request.“Additionally, we have provided the required statement in the submission system.
